# Mast cell‐deficient mice *Mcpt5Cre/Dicer* ^*fl/fl*^ redefine the role of mast cells in experimental bullous pemphigoid

**DOI:** 10.1002/ski2.70

**Published:** 2021-12-21

**Authors:** S. Nsiah‐Dosu, C. Scholz, Z. Orinska, C. D. Sadik, R. J. Ludwig, E. Schmidt, D. Zillikens, K. Hartmann

**Affiliations:** ^1^ Department of Dermatology University of Luebeck Luebeck Germany; ^2^ Department of Infectious Diseases and Microbiology University of Luebeck Luebeck Germany; ^3^ Division of Experimental Pneumology Research Center Borstel Leibniz Lung Center Borstel Germany; ^4^ Division of Experimental Pneumology, Research Center Borstel, Leibniz Lungenzentrum Airway Research Center North (ARCN) German Center for Lung Research (DZL) Borstel Germany; ^5^ Luebeck Institute of Experimental Dermatology (LIED) University of Luebeck Luebeck Germany; ^6^ Division of Allergy Department of Dermatology University of Basel Basel Switzerland; ^7^ Department of Biomedicine University of Basel Basel Switzerland

## Abstract

**Background:**

Bullous pemphigoid (BP) is the most frequent autoimmune blistering disease of the skin affecting the elderly. BP is immunopathologically characterized by autoantibodies against BP180 and BP230. With the growing evidence of cell‐mediated autoimmunity in the pathogenesis of BP, it still remains unclear whether mast cells (MCs) are involved, due to conflicting data obtained from Kit‐dependent MC‐deficient mouse models.

**Objectives:**

To clarify the role of MCs in experimental BP; the dynamics in cutaneous MC numbers, associated immune cells and the development of disease in Kit‐independent MC‐deficient mouse model.

**Methods:**

Employing a recently established murine adult passive transfer model of BP induced by the transfer of pathogenic immunoglobulin G (IgG), lesional skin biopsies were investigated histologically and immunohistochemically for the time‐dependent MC accumulation and dermal infiltration.

**Results:**

The numbers of cutaneous MCs increased following the induction of BP, in part, maintained by MC proliferation. Numbers of T cells, neutrophils and eosinophils in the skin also increased after BP induction, with eosinophils showing a preferential co‐localization with MCs. Furthermore, clinical disease manifestation in MC‐deficient *Mcpt5Cre/Dicer* ^*fl/fl*^ mice remained unchanged compared to MC‐sufficient *Dicer* ^*fl/fl*^ mice. The composition of the immune cell infiltration including as T cells, neutrophils and eosinophils was largely unaffected by the absence of MCs.

**Conclusion:**

MCs do not play a pivotal role in the pathogenesis of passive IgG‐transfer mediated BP model. Their increase in number may be a bystander effect following tissue injury. We therefore suggest caution regarding the selection of MCs as sole targets for the development of novel drugs for BP.

1

**What is already known about this topic?**

- There is much controversy surrounding the role of mast cells in bullous pemphigoid. Previous studies have employed the widely used Kit‐dependent mast cell‐deficient mouse strains, which are associated with several limitations.

**What does this study add?**

- We report for the first time the activity of mast cells in experimental bullous pemphigoid, using a Kit‐independent mast cell‐deficient mouse model.
- We describe the clinical inflammatory milieu orchestrated by dermal infiltrates in a time‐dependent manner, providing a better understanding of the disease pathogenesis.

## 2 INTRODUCTION

Bullous pemphigoid (BP) is an autoimmune skin blistering disease of the elderly, characterized by autoantibodies against two hemidesmosomal antigens BP180 and BP230 that adhere the epidermis to the dermis.^1^, ^2^ These autoantibodies cause loss of cell‐to‐matrix adhesion, resulting in subepidermal blistering.^2^, ^3^, ^4^

While anti‐BP180 autoantibodies have been noted to directly remodel hemidesmosomal function,^5^ skin pathology in BP is mostly driven by the induction of inflammation following the formation of immune complexes at the dermal‐epidermal junction (DEJ).^6^ Cell‐mediated autoimmunity is a significant contributor to the pathogenesis of BP.^7^, ^8^ Numerous studies have demonstrated the potential role of mast cells (MCs)^9^, ^10^, ^11^, ^12^, ^13^, ^14^ as well as the involvement of infiltrating cells including neutrophils,^8^ eosinophils^15^ and basophils^16^, ^17^ in the pathogenesis of BP. The accumulation of these cellular infiltrates in the skin and subsequent production of deleterious substances, such as reactive oxygen species and matrix metalloproteinases among others, compromise the function of the dermal‐epidermal adhesion complex, leading to the separation of DEJ.^18^, ^19^ However, the mechanisms that govern the infiltration and kinetics of inflammatory cells in tissue destruction are complex and have only lately begun to be more readily understood.

Research into the role of such cellular infiltrates has explored the function of MCs in the immunopathogenesis of BP. Since early observations of MCs within the lesional skin biopsies of BP patients,^9^, ^11^ disease model‐based studies of their functional role in BP and epidermolysis bullosa acquisita (EBA),^13^, ^20^ have led to conflicting results. Whether MCs are functionally involved in the pathogenesis of BP has remained unclear.

Until recently, studies investigating the function of MCs in various disease models have used *Kit*‐dependent MC‐deficient mouse strains.^13^ These strains, however, face some limitations such as anaemia and neutropenia in *Kit* ^*W/Wv*^ mice or neutrophilia and thrombocytosis in *Kit* ^*W‐sh/W‐sh*^ mice.^21^, ^22^ To overcome limitations associated with *Kit*‐dependent MC‐deficient mouse models, recent advances have led to the development of MC‐deficient mouse models independent of *Kit* ^23^, ^24^, ^25^, ^26^, ^27^ that, besides MC deficiency, have no other immune abnormalities, thus allowing the relevance of skin MCs to be studied.

To decipher the functional relevance of MCs in BP, we used a recently established a murine adult passive transfer model of BP^28^ and assessed cutaneous infiltration of MCs in conjunction with other immune cells. Moreover, we studied the development of experimental BP in *Kit*‐independent MC‐deficient *Mcpt5Cre/Dicer* ^*fl/fl*^ mice.^27^

## 3 MATERIALS AND METHODS

### 3.1 Mice

We used WT *C57BL/6J* mice^28^ and heterozygous *Mcpt5Cre* mice,^23^ which express Cre recombinase under control of the *Mcpt5* promoter, that were crossed with *Dicer* ^*fl/fl*^ C57BL/6J mice.^29^ As we and others have previously shown, *Mcpt5Cre/Dicer* ^*fl/fl*^ mice are characterized by selective constitutive ablation of connective tissue‐type MCs in various tissues including the back skin.^25^, ^27^ *C57BL/6J* mice were purchased from Janvier Labs (Le Genest‐Saint‐Isle, France). A total of 100 mice (43 females, 57 male) were used, all of which were aged between 6 and 13 weeks when used for experiments.

Mice were housed and bred at 22°C and 55% humidity, with a light‐dark cycle of 12 h, in specific pathogen‐free conditions and with ad libitum supply of food and water at the animal facility of the University of Luebeck, Luebeck, Germany. The experiments were performed in accordance with institutional and state guidelines on animal welfare, approved by the respective governmental administration and carried out by certified personnel.

### 3.2 Generation of anti‐COL17 IgG

The non‐collagenous region NC15A of murine type XVII collagen (COL17) was expressed as glutathione‐S‐transferase (GST) fusion protein, purified by affinity chromatography,^7^ and the generation of pathogenic anti‐COL17 immunoglobulin G (IgG) was performed as described.^30^ In brief, New Zealand white rabbits were immunized with recombinant GST‐tagged NC15A domain of murine COL17, the rabbit serum was purified using protein G, and the reactivity of IgG fractions was analyzed by indirect immunofluorescence microscopy of murine skin.^28^ Normal rabbit serum was obtained from C.C.Pro (Oberdorla, Germany).

### 3.3 Adult passive transfer BP mouse model

Rabbit anti‐COL17 IgG or normal rabbit IgG was injected *s.c*. at doses of 20 mg per injection into the neck of WT and *Mcpt5Cre/Dicer* ^*fl/fl*^ mice every second day over the course of 12 days (6 × 20 mg in total). Skin lesions (erythema, erosions and crusts) were assessed on days 0, 2, 4, 8 and 12. The percentage of the total body surface affected by lesions was calculated as described (ears, 5%; eyes, 1%; snout, 2.5%; forelegs, 10%; hind legs, 20%; tail, 10%; trunk, 40%; oral mucosa, 2.5%; and head and neck, 9%). At different time points, we obtained skin biopsies from the neck region for histological and immunohistochemical analyses.

### 3.4 Histology and immunohistochemistry

For the assessment of BP features, paraffin‐embedded sections from skin biopsies (5‐μm‐thick) were stained with haematoxylin and eosin. Demonstration of IgG deposition at the basement membrane was performed on frozen sections of lesional skin biopsies using immunofluorescence staining with fluorescein isothiocyanate (FITC)‐conjugated AffiniPure Donkey anti‐rabbit IgG (1:100; Jackson Immuno Research), incubated for 1 h at room temperature and washed three times for 5 min in phosphate‐buffered saline. Toluidine blue staining was performed on paraffin‐embedded lesional skin for the immunohistochemical identification of MCs, whilst the visualization of MCs with immunoflorescence used avidin (dilution 1:200; BioLegend), blocked with 3% bovine serum albumin (Carl Roth, Karlsruhe, Germany) in Tris‐buffered saline (TBS). To enumerate T cells, neutrophils and eosinophils, we stained sections with rat anti‐CD3 (clone CD3‐12, dilution 1:100; Abcam), anti‐Ly6G (clone 1A8, dilution 1:100; Abcam) and anti‐major basic protein (MBP) (dilution 1:250; purchased from Jacobsen Laboratory, Division of Allergy, Asthma and Clinical Immunology, Mayo Clinic), respectively. To assess proliferation of MCs, sections were double stained with anti‐Ki67 (clone 16A8, dilution 1:100; BioLegend). Prior to incubation with antibodies, we performed antigen retrieval using citrate buffer at pH 6.0 (anti‐CD3 and anti‐Ki67) or enzymatic digestion (anti‐Ly6G and anti‐MBP; Digest‐All Pepsin; Life Technologies). All primary antibodies were diluted in TBS with normal goat serum (2.5%) and applied overnight. Corresponding isotype controls (rat IgG1, IgG2a; BioLegend) for all primary antibodies were applied in relation to their respective dilutions as indicated above. Alexa Fluor® 594‐coupled goat anti‐rat antibody was used as secondary antibody (dilution 1:200; Jackson ImmunoResearch). Histologic and immunoflorescence analyses were performed at 200× magnification in five high power fields. Counts were used to calculate the mean number of cells per mm^2^ using a BZ‐9000E series Keyence microscope with BZ Analyzer software (Keyence, Neu‐Isenburg) and Image J software 1.52q (National Institute of Health). The degranulation of MCs was assessed semiquantitatively according to Wershil et al.^31^ with slight modifications. MCs were classified as not degranulated (intact) and degranulated (>10% of the granules exhibiting fusion or discharge).

### 3.5 Statistical analysis

Statistical analysis was performed, as indicated in the figure legends, with one‐way analysis of variance (ANOVA) with Dunn's multiple comparisons test, two‐way ANOVA with Sidak's and Tukey's multiple comparisons test as well as with Welch's *t*‐test using GraphPad Prism 8 (GraphPad Software). In all tests, a *p‐value* of 0.05 was considered to be statistically significant.

## 4 RESULTS

### 4.1 Passive transfer of anti‐COL17 IgG in adult mice produces clinical and immunopathological features of the human disease

**FIGURE 1:**
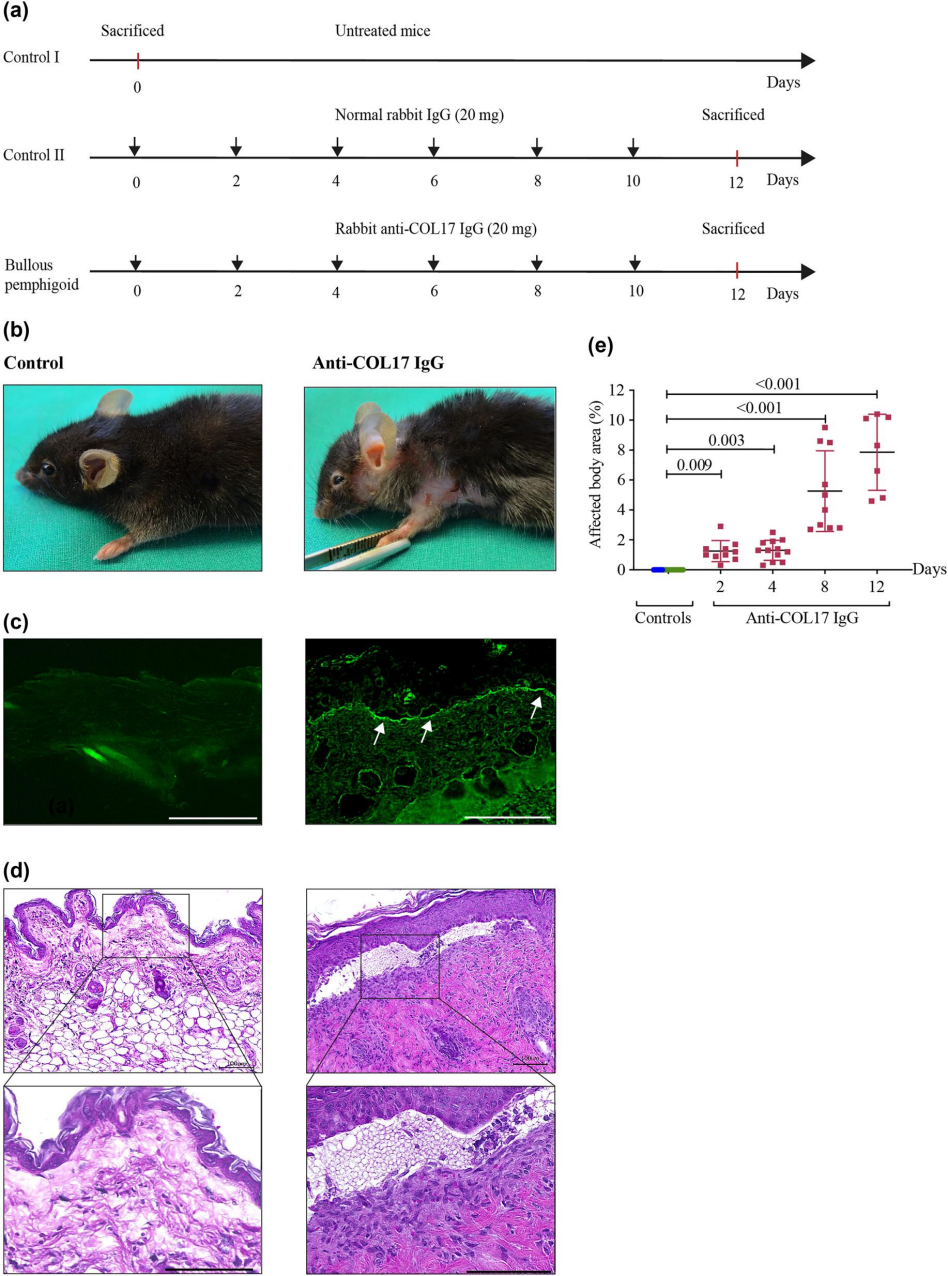
Experimental set‐up of passive transfer mouse model of bullous pemphigoid (BP) in adult mice. (a) Experimental BP was induced by *s.c*. injections of anti‐COL17 immunoglobulin G (IgG) on every second day over the course of 12 days (lower line; BP). Control mice were either not treated (upper line; control I) or treated with normal rabbit IgG over the course of 12 days (mid line; control II). (b–d) Representative photographs of the BP mouse phenotype. (b) Skin phenotypes on day 12 (left panel: control II; right panel: anti‐COL17 IgG‐treated mouse). Lesions developing in anti‐COL17 IgG‐treated mice are characterized by erythema, erosions and crusts. (c) Immunofluorescence staining with fluorescein isothiocyanate‐conjugated AffiniPure Donkey anti‐rabbit IgG of the dermal‐epidermal junction (DEJ) in normal rabbit IgG‐treated mouse (left panel) and lesional skin biopsy from anti‐COL17 IgG‐treated mouse (right panel) on day 4 (magnification: 400×, scale bar: 100 μm). White arrows indicate the deposition of IgG autoantibodies at the DEJ. (d) Haematoxylin and eosin staining of a skin biopsy obtained from control II (left photograph) and a lesional skin biopsy from anti‐COL17 IgG‐treated mouse on day 12 (right photograph). The photographs show the separation of the DEJ, thickened epidermis and dermal infiltration (upper panels, magnification: 200×, scale bar: 100 μm; lower panels, magnification: 400×, scale bar: 100 μm). (e) Time‐dependent development of BP lesions was scored by calculating the affected body surface as described.^28^ Control mice (controls) included control I (*n* = 12, green dots) and control II (*n* = 6, blue dots). Statistical analysis was performed using one‐way analysis of variance with Dunn's multiple comparisons test. Data are presented as a scatter diagram with mean ± SD

In line with previous results,^28^, ^32^, ^33^ we observed the induction of experimental BP by repetitive injections of anti‐COL17 pathogenic IgG into WT *C57BL/6J* mice (Figure 1). During BP induction, mice developed erythema and erosions on the ears, the neck, limbs, trunk, and around the eyes (Figure 1b). The disease severity in BP mice clearly increased with the progression of the disease (Figure 1e). Clinical disease manifestation was associated with IgG deposition along the DEJ (Figure 1c), subepidermal blistering, as well as a dermal leukocyte infiltration (Figure 1d).

### 4.2 Numbers of MCs are increased in experimental BP skin lesions

**FIGURE 2:**
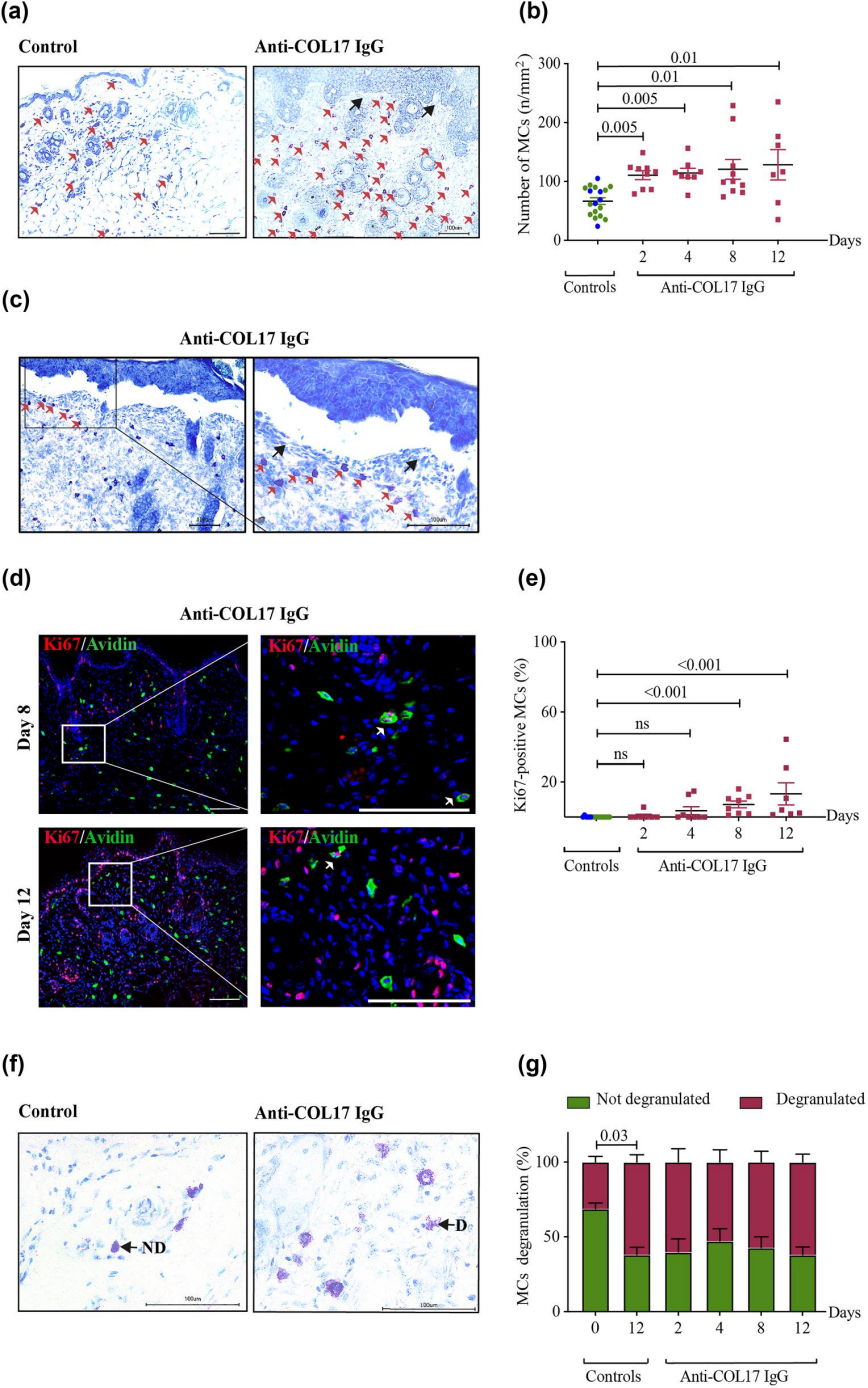
Cutaneous mast cells (MCs) increase in number and proliferate during experimental bullous pemphigoid (BP). (a–c) Increase in MC numbers as well as their preferential localization in experimental BP. (a, c) Red arrows indicate MCs and black arrows indicate separation of the dermal‐epidermal junction (a, magnification: 200×, scale bar: 100 μm; c, left image, magnification: 200×, scale bar: 100 μm; right image, magnification: 400×, scale bar: 100 μm). (d, e) Proliferation of MCs in experimental BP. (d) White arrows indicate double‐positive proliferating MCs (left panel, magnification: 200×, scale bar: 100 μm; right panel, magnification: 600×, scale bar: 100 μm). MCs were visualized using double immunofluorescence staining with avidin and anti‐Ki67. (f, g) Degranulation of MCs in experimental BP. (f) Black arrows indicate not degranulated and degranulated (d) MCs (magnification: 600×, scale bar: 100 μm). (a, c, f) MCs were visualized using toluidine blue staining. (b, e, g) Statistical analyses were performed using (b, e) one‐way analysis of variance (ANOVA) with Dunn's multiple comparisons tests and (g) two‐way ANOVA with Tukey's multiple comparisons test. Data are presented as (b, e) scatter diagrams with mean ± SEM and (e) as bar graph with mean ± standard error of mean (SEM). Control mice (controls) include control I (*n* = 12, green dots) and control II (*n* = 6, blue dots). (g) At each time point, six to eight mice were investigated

Following the induction of BP, we observed a significant increase in MC numbers as early as day 2 (Figure 2). Increased MC numbers remained elevated over the entire course of the experiment (Figure 2a,b). MCs were found to cluster particularly beneath the separated DEJ (Figure 2c). Despite the increase in MC numbers on day 2 after the initial injection of anti‐COL17 IgG and the persistence of elevated numbers of MCs with increasing disease activity, no correlation was observed between the number of MCs and disease activity (data not shown). This suggests that MCs are not the main drivers of the disease. The progressive increase of MCs and their preferential localization beneath the separated DEJ could be a bystander effect.

### 4.3 Proliferation of MCs is increased in late experimental BP

To explore the origin of increased MC numbers, we performed double immunofluorescence staining of MCs with avidin‐FITC and Ki67‐specific antibodies (Figure 2d,e). There were only few proliferating MCs detectable in the BP mouse skin samples on day 2, comparable to the numbers we detected in the samples of our controls. This indicates that mechanisms other than proliferation, such as extravasation or tissue redistribution, may primarily account for the early increase of MCs. On the other hand, pronounced proliferation could be observed on days 8 and 12, after multiple injections of pathogenic anti‐COL17 IgG. This suggests that proliferation is involved in maintaining increased MC numbers at later stages of the BP disease process.

### 4.4 MC degranulation is not specific to anti‐COL17 IgG treatment

We detected MC degranulation in anti‐COL17 IgG‐treated mice in situ upon injection of antibodies (Figure 2f,g). However, there was no significant difference between anti‐COL17 IgG‐treated mice and control mice which received normal rabbit IgG (Figure 2g). This suggests that MC degranulation is not specific to anti‐COL17 IgG but could rather be induced by repeated *s.c*. injections or provoked by scratching. Such mechanically induced MC degranulation has been reported by Xanthos et al.^34^

### 4.5 Numbers of T cells, neutrophils and eosinophils are increased in experimental BP skin lesions

**FIGURE 3:**
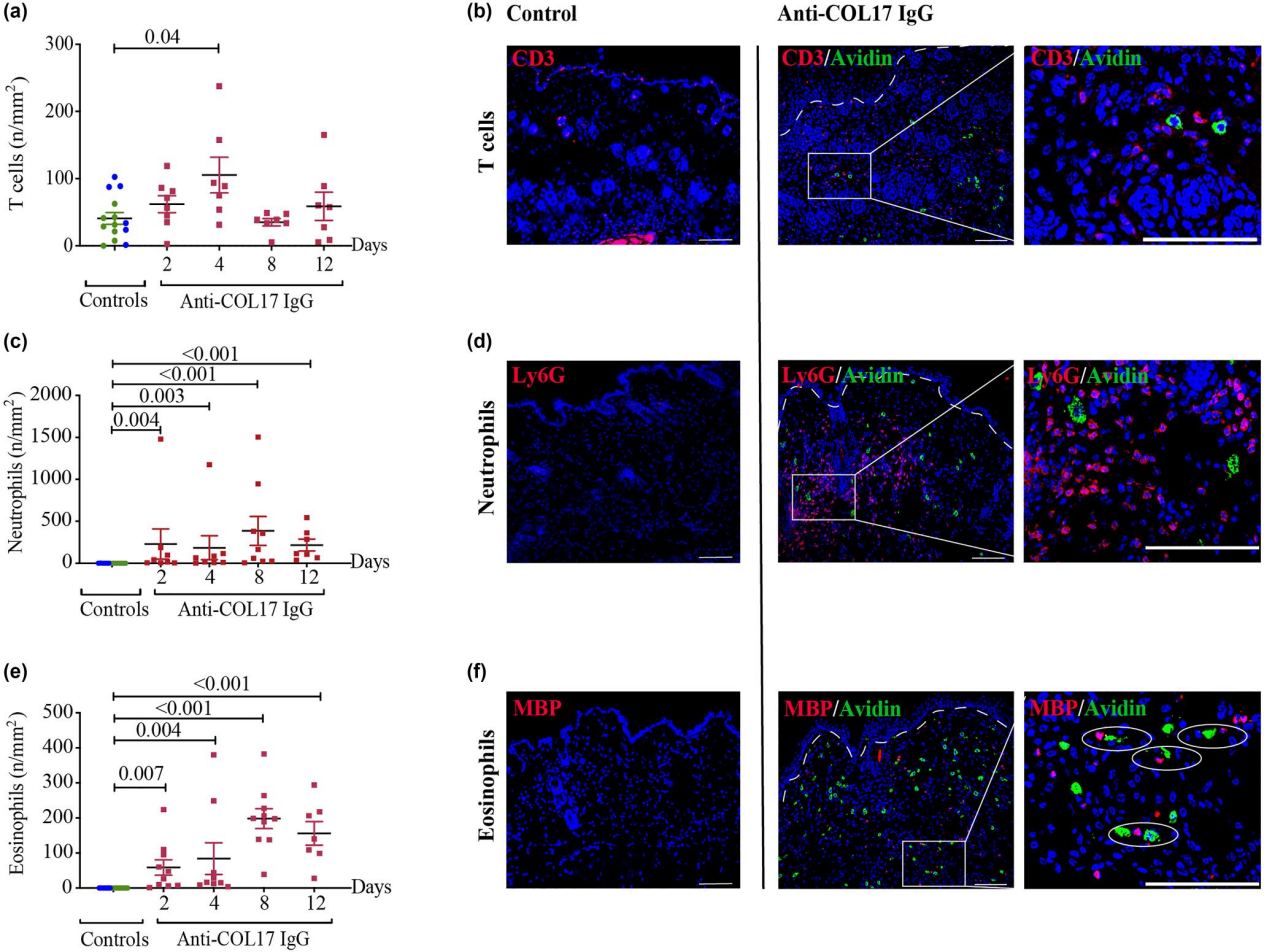
Numbers of T cells, neutrophils and eosinophils are increased in experimental bullous pemphigoid (BP) skin lesions. Lesional skin biopsies of anti‐COL17 immunoglobulin G‐treated mice were analyzed for (a, b) CD3‐positive T cells, (c, d) Ly6G‐positive neutrophils and (e, f) major basic protein‐positive eosinophils using immunofluorescence staining. (a, c, e) Statistical analysis was performed using one‐way analysis of variance with Dunn's multiple comparisons test. Data are presented as scatter diagrams with mean ± standard error of mean. Control mice (controls) include control I (*n* = 8, green dots) and control II (*n* = 6, blue dots). (b, d, f) Infiltrating of T cells on day 4, neutrophils on day 8 and eosinophils on day 12 are depicted using double immunofluorescence staining with avidin‐positive mast cells (MCs; middle panels, magnification: 200×, scale bar: 100 μm; right panels, magnification: 600×, scale bar: 100 μm). (f) Circles (right photograph) indicate co‐localization of eosinophils and MCs

To explore the involvement of other inflammatory cells during the development of experimental BP, we next determined the numbers of T cells, neutrophils and eosinophils in the skin of WT *C57BL/6J* mice during the development of the BP phenotype over 12 days (Figure 3). By comparison of anti‐COL17 IgG‐treated mice with the controls, we found significantly higher numbers of T cells (Figure 3a,b), neutrophils (Figure 3c,d) and eosinophils (Figure 3e,f) in anti‐COL17 IgG‐treated mice, which confirms previous observations in humans^6^ as well as this and other mouse models of BP,^4^, ^13^, ^28^, ^33^ indicating that the passive transfer model used in this study is suitable for the investigation of immune cells in BP. The numbers of neutrophils and eosinophils both increased at the beginning and in the late phase of disease (Figure 3c,e), possibly induced by the repeated injections of anti‐COL17 IgG, which may represent acute and chronic stages of inflammation. Interestingly, double staining with avidin‐positive MCs revealed that eosinophils (Figure 3f), unlike neutrophils (Figure 3d), often co‐localize with MCs, which suggests a possible cross‐talk between these two cell types.

### 4.6 Constitutive deficiency of MCs in *Mcpt5Cre/Dicer* ^*fl/fl*^ mice does not alter the development of experimental BP

**FIGURE 4:**
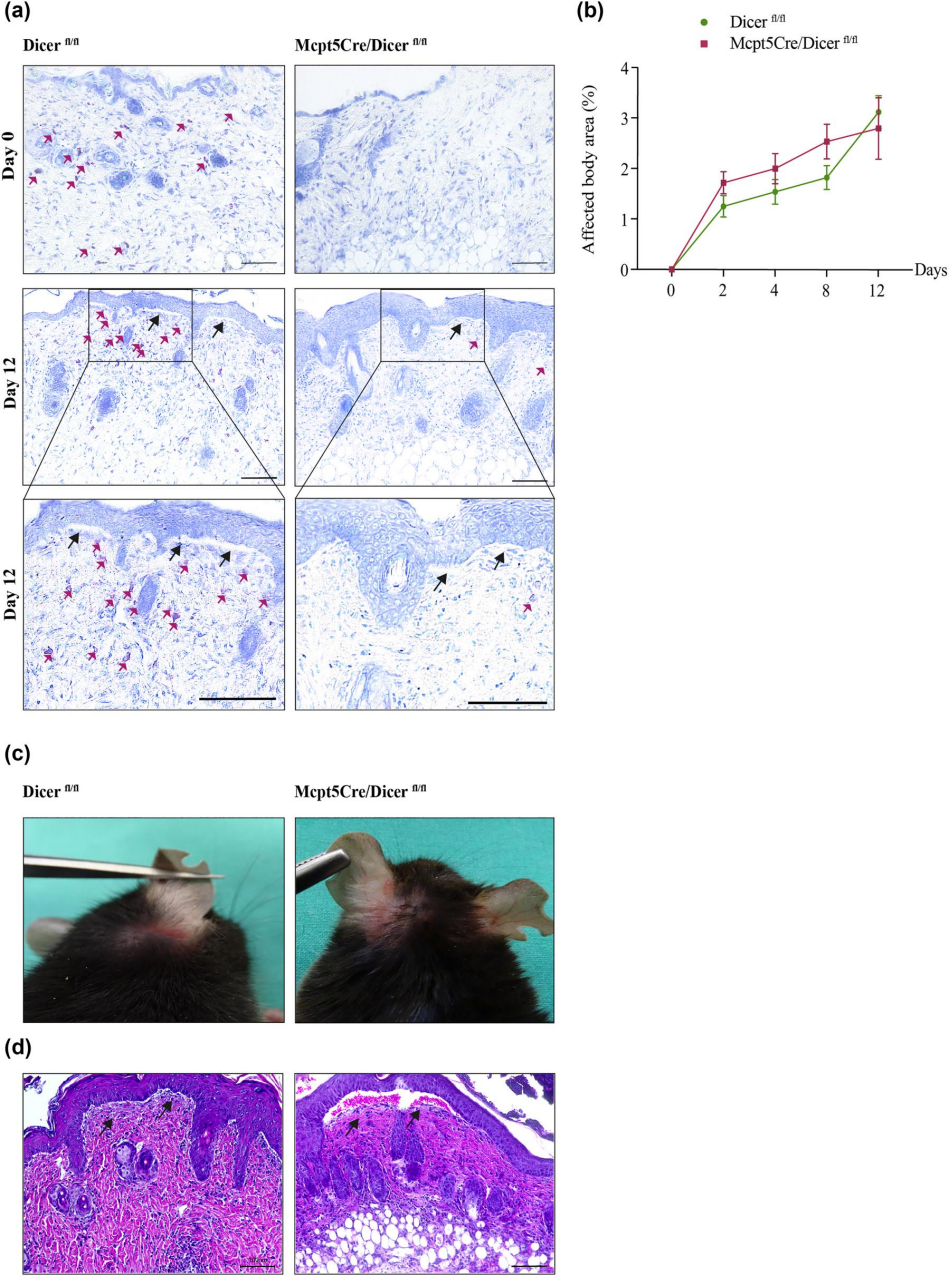
Constitutive deficiency of mast cells (MCs) in *Mcpt5Cre/Dicer* ^*fl/fl*^ mice does not alter the development of experimental bullous pemphigoid (BP). (a–d) Development of the BP phenotype in MC‐deficient *Mcpt5Cre/Dicer* ^*fl/fl*^ and MC‐sufficient *Dicer* ^*fl/fl*^ control mice. (a) To confirm the absence of MCs in *Mcpt5Cre/Dicer* ^*fl/fl*^ mice, histological staining of MCs with toluidine blue before and after the induction of BP was performed. Red arrows indicate MCs (upper and middle images, magnification: 200×, scale bar: 100 μm; lower images, magnification: 400×, scale bar: 100 μm). Black arrows indicate the separation of the dermal‐epidermal junction (DEJ). (b) Severity of the BP phenotype in *Mcpt5Cre/Dicer* ^*fl/fl*^ *and Dicer* ^*fl/fl*^ mice was assessed at different time points by calculating the affected body surface. At each time point, 6–19 mice were examined. Data were analyzed with two‐way analysis of variance with Sidak's multiple comparisons test. Data are presented as a curve diagram with mean ± standard error of mean. (c) Clinical manifestation of BP in *Mcpt5Cre/Dicer* ^*fl/fl*^ mice and *Dicer* ^*fl/fl*^ mice. (d) Representative photographs of histological staining (haematoxylin and eosin) of the head and neck lesional skin in MC‐deficient *Mcpt5Cre/Dicer* ^*fl/fl*^ and MC‐sufficient *Dicer* ^*fl/fl*^ control mice. Black arrows indicate the separation of the DEJ (magnification: 200×, scale bar: 100 μm)

Prompted by the above findings, we explored whether MCs play an active functional role in BP. For this purpose, we induced experimental BP in constitutively MC‐deficient *Mcpt5Cre/Dicer* ^*fl/fl*^ mice (Figure 4). Since repopulation of MCs has been reported in *Kit* ^*W‐sh/W‐sh*^ mice during inflammation,^12^, ^21^ we assessed MC numbers at different time points over the course of the experiment and were able to exclude repopulation of MCs in *Mcpt5Cre/Dicer* ^*fl/fl*^ mice on days 8 and 12 (Figure 4a; data not shown). Following the induction of BP, we found no significant difference in disease severity between *Mcpt5Cre/Dicer* ^*fl/fl*^ and *Dicer* ^*fl/fl*^ mice (Figure 4b). Both strains exhibited comparable clinical and histologic features, such as blisters, separation of DEJ, thickened epidermis as well as a dense cellular infiltrate in the upper dermis (Figure 4c,d), despite the near complete absence of MCs in *Mcpt5Cre/Dicer* ^*fl/fl*^ mice.

### 4.7 Constitutive deficiency of MCs in *Mcpt5Cre/Dicer* ^*fl/fl*^ mice does not affect numbers of T cells, neutrophils and eosinophils

**FIGURE 5:**
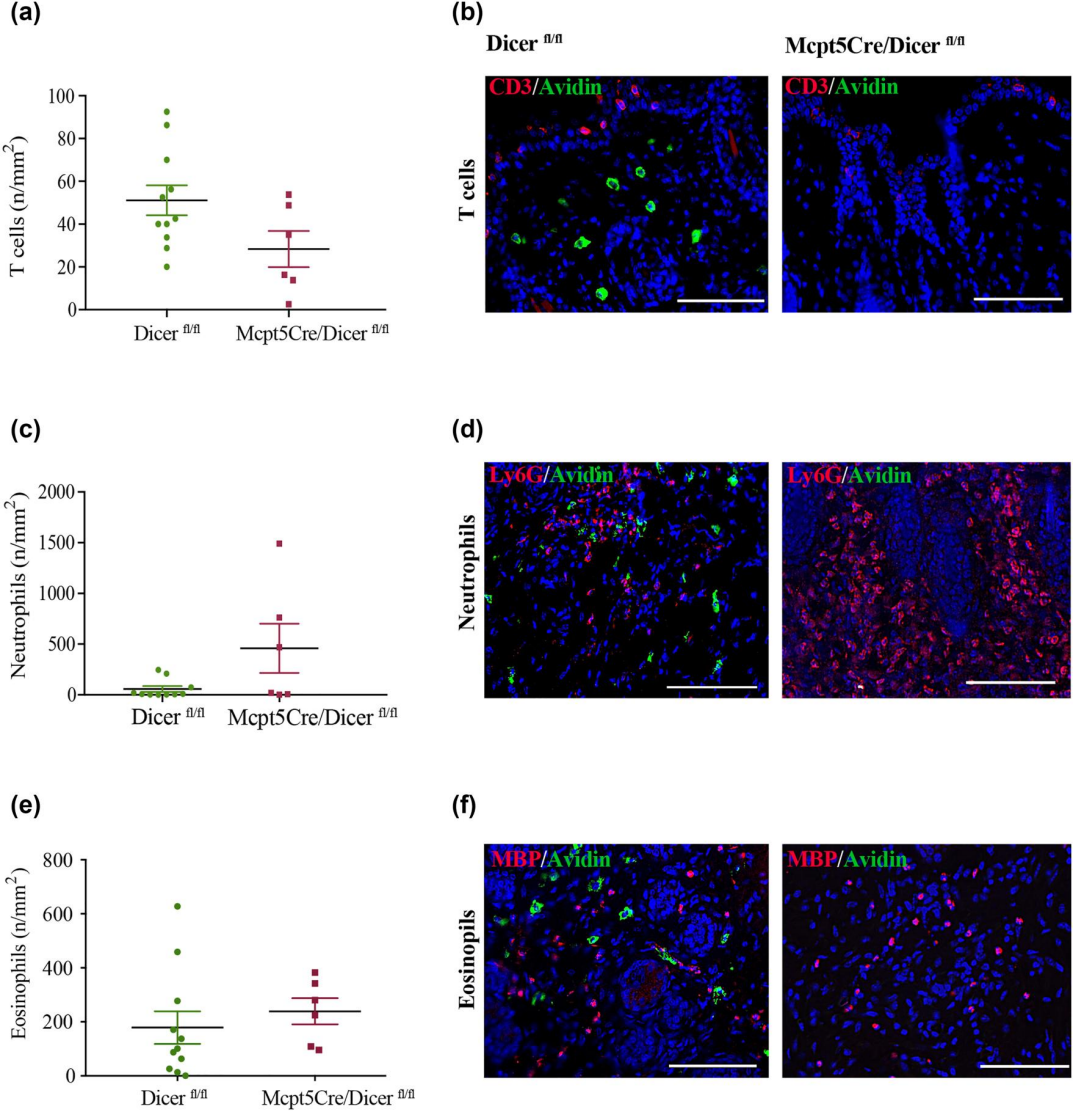
Constitutive deficiency of mast cells (MCs) in *Mcpt5Cre/Dicer* ^*fl/fl*^ mice does not alter the numbers of T cells, neutrophils and eosinophils in experimental bullous pemphigoid (BP). Lesional skin biopsies of anti‐COL17 immunoglobulin G‐treated MC‐deficient *Mcpt5Cre/Dicer* ^*fl/fl*^ mice and MC‐sufficient *Dicer* ^*fl/fl*^ control mice were analyzed for (a, b) CD3‐positive T cells, (c, d) Ly6G‐positive neutrophils and (e, f) major basic protein‐positive eosinophils using immunofluorescence staining on day 12. (a, c, e) Statistical analysis was performed with Welch's *t*‐test (no statistically significant differences were observed). Data are presented as scatter diagrams with mean ± standard error of mean. (b, d, f) Infiltrates of T cells, neutrophils and eosinophils on day 12 are depicted using double immunofluorescence staining with avidin‐positive MCs (magnification: 400×, scale bar: 100 μm)

Considering the importance of cell‐mediated autoimmunity to the pathogenesis of BP,^7^, ^8^ it is believed that some of these cellular infiltrates, such as neutrophils, depend on MCs for their recruitment.^13^ In order to investigate whether MC deficiency in experimental BP affects other immune cells, we evaluated the numbers of T cells, neutrophils and eosinophils in *Mcpt5Cre/Dicer* ^*fl/fl*^ and *Dicer* ^*fl/fl*^ mice on day 12 of our experiment (Figure 5). There were no significant differences in T cell (Figure 5a,b), neutrophil (Figure 5c,d) and eosinophil numbers (Figure 5e,f). This observation supports the notion that the absence of MCs in experimental BP is perhaps compensated by other immune cells with overlapping roles, highlighting that the recruitment of neutrophils in experimental BP can occur in a MC‐independent manner.

## 5 DISCUSSION

The potential role of MCs in the pathogenesis of BP has been the subject of debate in recent times. Although increased numbers of MCs have been reported in a variety of human pathologies^35^, ^36^, ^37^, ^38^, ^39^, ^40^, ^41^, ^42^, ^43^ and disease models,^44^, ^45^, ^46^, ^47^, ^48^ in BP, the concept of increased MC numbers and MC degranulation was first reported as early as 1978 by Wintroub and colleagues.^9^ To this day, however, the functional relevance of MCs in the pathogenesis of BP has remained controversial. This comes after one of the first murine studies using *Kit‐*dependent MC‐deficient mice reported protection from BP in these mice,^13^ but later work by Kasprick et al., which employed the *Kit‐*independent *Mcpt5Cre/iDTR* ^*fl/fl*^ strain in their study of MCs in the a model of EBA contradicted this finding.^20^ Yet, while both EBA and BP are often placed in the same context, they are not only different in regard to target autoantigens,^1^, ^49^ but also with respect to their different pathologies and prognosis.

To address the role of MC in BP, we herein specifically selected *Mcpt5Cre/Dicer* ^*fl/fl*^ mice because this strain holds several advantages compared to other MC‐deficient *Mcpt5Cre/iDTR* ^*fl/fl*^ mice. The *Mcpt5Cre/iDTR* mouse strain relies on selective expression of a high‐affinity receptor for diphtheria toxin (DT) in MCs and its targeting by DT treatment.^23^, ^50^ Previous studies investigating other inflammatory disease models in *Mcpt5Cre/iDTR* ^*fl/fl*^ mice have shown some repopulation of cutaneous and peritoneal MCs about 3 weeks after the last DT treatment,^51^, ^52^ which raises concern on the sufficiency of MC depletion for the study of autoimmune blistering diseases. Furthermore, 4 weeks of DT pretreatment, followed by 12 additional days of passive anti‐COL17 IgG injection into *Mcpt5Cre/iDTR* ^*fl/fl*^ mice is a lengthy experimental time that may put mice under stress, which in turn, influences MCs.^53^ For this reason, *Mcpt5Cre/Dicer* ^*fl/fl*^ mice were employed to overcome these limitations.

Interestingly, in our *Kit*‐independent MC‐deficient *Mcpt5Cre/Dicer* ^*fl/fl*^ mice, experimental BP developed independently of MCs. Following the induction of BP, we observed a separation of the DEJ regardless of the absence of MCs. This finding is in contrast to previous studies reporting on protection from BP in *Kit‐*dependent MC‐deficient *Kit* ^*W/Wv*^ and *Mgf* ^*Sl/Sl‐d*^ mice.^13^, ^21^ These previous studies, however, employed a passive transfer model of BP in neonatal mice lasting only for 12–24 h, which limits the comparability to our results. However, our study ties in with recent reports on unaltered tumour growth^54^ and wound healing^55^ in *Kit*‐independent MC‐deficient mice, despite pronounced MC infiltration. Furthermore, in our *Mcpt5Cre/Dicer* ^*fl/fl*^ mice, the composition of the immune cell infiltrate in the skin turned out to be largely unaffected by the absence of MCs. This implies that the absence or presence of MCs in experimental murine BP is not essential to drive skin pathology. The recruitment of neutrophils can be mediated in a MC‐independent manner.^14^ Again, these findings contrast with previous observations made in *Kit*‐dependent MC‐deficient mice. For example, Chen et al. found that in *Kit*‐mutant mice, anti‐BP180 antibody‐induced neutrophil infiltration depends mainly on MCs.^13^ Thus, our results, contradicting previous observations both on BP development as well as MC‐dependent immune cell infiltration in *Kit*‐dependent MC‐deficient mice, highlight the importance of a careful interpretation of data relying on *Kit*‐dependent MC‐deficient mouse models.

## CONFLICT OF INTEREST

The authors confirm that no conflict of interest to declare.

## Data Availability

The data that support the findings of this study are available from the corresponding author upon reasonable request.
